# Mathematical modeling of peritoneal buffer transport and acidosis correction in patients on peritoneal dialysis

**DOI:** 10.1038/s41598-026-53800-0

**Published:** 2026-07-04

**Authors:** M. Pietribiasi, J. Stachowska-Pietka, J. Waniewski, B. Lindholm, O. Heimbürger

**Affiliations:** 1 Nalecz Institute of Biocybernetics and Biomedical Engineering, Polish Academy of Sciences, Warsaw, Poland; 2 Renal Medicine, Department of Clinical Science Intervention and Technology, Karolinska Institutet, Stockholm, Sweden

**Keywords:** Peritoneal transport, Mathematical modeling, Buffer base, Bicarbonate, Lactate, Engineering, Nephrology

## Abstract

Assessing buffer base kinetics in dialysate using the three-pore model of peritoneal transport is not enough to predict changes to the acid-base equilibrium in blood during peritoneal dialysis (PD). We complemented the three-pore model with a model of acid-base homeostasis to evaluate differences between PD sessions using dialysis fluid buffered with lactate (PD4) or bicarbonate/lactate (B/L). Six patients underwent two 4-hour dwells with either B/L or PD4; blood and peritoneal fluid sampling was used to quantify intraperitoneal volume—using a radioiodinated albumin as volume marker—and solute concentrations. The integrated model describes CO_2_ and O_2_ transport across central circulation and tissues, predicting changes in acid-base equilibrium driven by peritoneal transport of bicarbonate, lactate, and dissolved CO_2_. The model accurately predicted dialysate concentrations of bicarbonate and dissolved CO_2_, with errors in the range 5–10% of measured values for both fluids, and with similar prediction errors for plasma concentrations despite tuning the model to dialysate data only. Estimated transport parameters fell into the expected ranges and were generally comparable between fluids. These results validate our understanding of transport kinetics and acid-base homeostasis during PD, demonstrating that different buffer compositions do not appear to impact small solute transport nor mechanisms of acid-base regulation.

**Supplementary Information:**

The online version contains supplementary material available at 10.1038/s41598-026-53800-0.

## Introduction

Acid-base correction is a critical component of the management of patients with kidney failure including those undergoing peritoneal dialysis (PD). In PD, peritoneum serves as a natural dialysis membrane, allowing exchange of solutes and fluids between the dialysate and the bloodstream. This process is essential for maintaining homeostasis, particularly in the context of metabolic acidosis, which is prevalent among patients with renal failure due to the impaired acid excretion, reduced bicarbonate regeneration, accumulation of uremic toxins and other factors^1,2^. The treatment of acidosis in PD involves the addition to the dialysis fluid of basic buffers such as bicarbonate, lactate and acetate, which diffuse from the peritoneal cavity to the sub-peritoneal capillaries, replenishing the natural buffer systems in the body fluids. Effective acid-base management has a direct impact on patient morbidity and mortality, as well as the overall quality of life^1–3^. In contrast to hemodialysis (HD), where solute transport is determined by the treatment settings, peritoneal dialysis involves dynamic changes in dialysate composition during the dwell, making the assessment of solute transport more complex. This is especially true for buffers, which undergo chemical conversion in the body, making mass balance estimations less straightforward.

Traditional peritoneal lactate-buffered dialysis fluids with low pH (5.2–5.5) are associated with adverse local effects on the peritoneum due to their content of glucose degradation products (maily reactive aldehydes) which contribute to the formation of advanced glycation end-products (AGE) in the peritoneum. Therefore, more biocompatible solutions, with pH closer to physiological values, and with other buffers than only lactate, have been developed. Multi-compartment bag systems, using pure bicarbonate as a buffer (Bicavera^®^) or a mix of bicarbonate and lactate (Physioneal^®^), or lactate (Balance^®^) were shown to be effective in terms of management of acidosis and were able to better preserve peritoneal membrane health^3^. However, the presence of two buffer bases makes quantifying total alkali transport more challenging, as also shown in HD by Leypoldt et al.^4^.

Mathematical models, such as the three-pore model of the peritoneal membrane, have played a significant role in understanding the kinetics of solute transport during PD^5–7^. These studies provided valuable insights into how different dialysis regimens can be tailored to optimize the removal of excess water and uremic toxins, emphasizing the need for individualized treatment based on patient-specific factors. While recently we saw a surge in interest in modeling acid-base homeostasis during HD^8–10^, only a few attempts have been made for PD^11^. We previously developed and validated a mathematical model describing acidosis correction and acid-base equilibrium during intermittent HD^12,13^. Although the mechanisms involved in acid-base homeostasis are not treatment-specific, the continuous nature of the PD treatment and the different magnitude of the forces involved during peritoneal exchanges leaves questions as to whether the same description of these phenomena can be employed in both scenarios, and if there are factors that are more important during PD than HD.

In this paper, we describe the results of computational simulations of the outcome of peritoneal dialysis dwells with two different dialysis buffers, adapting our mathematical model of acid-base equilibrium during HD and integrating it with the description of transperitoneal transport of buffer base according to the three-pore theory. Our aim is to advance our understanding of the physiology underlying the complex mechanisms of acid-base homeostasis at work in shaping the body response to the supplementation of buffer during dialysis.

## Methods

### Clinical data

The data used in this study were previously partially published, in aggregated form, by Heimburger and Mujais^14^. Seven patients were included in the study. One patient was removed from the analysis because of insufficient drainage before one dwell (residual volume over 1 L). Thus, data from 6 patients (2 females, average body weight 74.8 ± 15.3 kg)^14^ were used in this study to fit the mathematical model. Each patient underwent two 4-hour dwells in consecutive weeks with 2 L of glucose 3.86% solution and two different dialysis buffers: a mix of bicarbonate and lactate (Physioneal, from here on called “B/L”; Baxter Healthcare, Castlebar, Ireland) and pure lactate (Dianeal PD4, from here on called “PD4”; Baxter Healthcare, Castlebar, Ireland). Radio-iodinated human serum albumin (RISA) was used as volume marker for the assessment of residual volume, peritoneal absorption, and changes of intraperitoneal volume during the dwell. The composition of the residual fluid in the peritoneal cavity was assessed based on the sample taken from spent dialysate before the start of the dwell. Intraperitoneal fluid was sampled frequently during the dwell, at t = 0, 3, 6, 10, 15, 20, 25, 30, 40, 50, 60, 90, 120, 180, and 240 min after the fluid infusion. The volume of the samples was 16, 14, 8, 8, 8, 8, 14, 8, 8, 14, 8, 14, 8, and 22 mL, respectively. Dialysate bags were weighted to assess total fluid removed and infused. The weight of the bags was corrected for the different weight of the different plastic bags. Samples of venous blood were drawn from the patient before the start of the dwell and at t = 15, 60, 120, 240 min. For both dialysate and blood samples, laboratory analysis was employed to measure the concentrations of urea, glucose, creatinine, sodium, potassium, chloride, ionic phosphate, lactate concentrations using a Monarch 1000 automatic centrifugation analyzer (Instrumentation laboratories, Inc.,Lexington, MA, USA); a blood gas analyzer (AVL OMNI Combi Analyzer, AVL List BmbH Medizintechnik, Graz, Austria) was used to assess the acid-base equilibria in the samples (bicarbonate concentration, pH in plasma, partial pressure of CO_2_, hematocrit) and pH in the dialysate was measured by a pH meter. The concentration of dissolved CO_2_ in plasma and dialysate fluid was obtained multiplying the partial pressure of CO_2_ by its solubility in water, 0.23 mmol/L/mmHg. Creatinine measurements were corrected for glucose concentration^15^; plasma concentrations were corrected for plasma water fraction^16^. The parameters describing blood composition were similar before the dwell studies with B/L and PD4 solutions.

No significant difference was found in the residual volume remaining after the exchanges preceding the dwell with the two fluids (466 ± 160 mL before B/L, and 316 ± 74 mL before PD4; *p* > 0.05). There was a statistically significant difference in infused volumes, amounting on average to 30 mL. Peritoneal absorption rate was calculated separately for each dwell from the disappearance of the volume marker, as described in^15,17^, and was 3.66 ± 1.48 mL/min for B/L and 2.79 ± 1.02 mL/min for PD4 (*p* = 0.11). In general, no difference in solute concentrations between the two PD solutions was found at the start of the peritoneal dwell, except for the expected differences in lactate, bicarbonate and dissolved CO_2_.

The initial (after 3 min from the end of dialysate infusion) lactate concentration in the peritoneal fluid was 15.2 ± 0.4 mmol/L in B/L and 38.1 ± 1.5 mmol/L in PD4; bicarbonate concentration was 25.4 ± 1.1 mmol/L in B/L and 3.4 ± 1.1 mmol/L in PD4; dissolved CO_2_ concentration was 1.30 ± 0.08 mmol/L in B/L and 0.34 ± 0.04 mmol/L in PD4 (all with *p*-value < 0.05 between fluids). The only other significant difference in dialysate composition at 3 min was in sodium, which was 1.3% higher in PD4 compared to B/L.

The study was conducted in adherence to the Declaration of Helsinki. All methods were carried out in accordance with relevant guidelines and regulations. The study and all experimental protocols were approved by the Ethics Committee of the Karolinska Institute (EPN) at the Karolinska University Hospital Huddinge, Stockholm, Sweden. Informed consent was obtained from all participants before any study procedures were performed.

### Mathematical model

The model (3PM/ABE) is comprised of two coupled components, a model of peritoneal transport of water and solutes (3PM), and a model of acid-base equilibrium in the body (ABE). All simulations were carried out assuming that the initial conditions in plasma corresponded to the pre-dialysis measurements, while in dialysate initial conditions corresponded to the values measured 3 min after the end of the fluid infusion.

The peritoneal transport of water and solutes across the peritoneal membrane was described using the extended three-pore model, accounting for the effect of vasodilation on peritoneal transport parameters^18–21^. This approach has been extensively described in literature, and its assumptions will only be briefly summarized here. The passage of molecules take place across cylindrical, uniformly shaped and distributed pores in the capillary wall, belonging to one of three categories: large pores (LP), through which fluid, small solutes (e.g. urea, ions) and large solutes (e.g. albumin) can be transported; small pores (SP), which restrict the passage of large solutes but not of small ones; ultrasmall pores (UP), which allow free water transport but deny the passage of solutes, and are assumed to represent transport through aquaporins^22^. Water transport is driven by the interplay of osmotic, oncotic and hydraulic pressure gradients in peritoneal fluid and plasma. Solutes are transported both by convection and by diffusion, following the concentration gradients. Peritoneal absorption was assumed to be constant and equal to the rate calculated from the volume marker.

We used standard three-pore model (3PM) parameters, reported in Appendix A (Supplementary Material), except for those that were directly calculated from the data (e.g. peritoneal absorption) or estimated to fit the model to the measurements. Dialysate concentrations were determined by the 3PM equations, while the acid-base model simulated the changes in plasma bicarbonate and CO_2_ concentrations. The plasma concentrations of all other solutes were assumed to be known inputs of the model; their profiles were calculated by linearly interpolating between the measured values.

The acid-base model, originally developed for HD, was adapted from the work of Rees and Andreassen^23,24^ and described in detail and validated for HD in previous publications^12,13^. The model predicts the acid-base status of four body compartments: lung capillaries and alveoli, arterial blood pool, mixed-venous blood pool, and tissues (comprising interstitial and intracellular spaces). Each compartment is defined by ordinary differential equations describing the evolution in time of several variables. These are: the fractions of expired O_2_ and CO_2_ gas in the lung compartment; plasma volume, base excess, total CO_2_ and O_2_ concentrations in the arterial and mixed-venous blood compartments; interstitial fluid volume, and total CO_2_ and O_2_ concentrations in the tissue compartment. The variables tracked in each compartment are used to solve nonlinear systems of equations that describe the acid-base biochemistry, allowing to calculate other derivate quantities such as, among others, plasma and erythrocyte bicarbonate concentrations, partial pressures of CO_2_ and O_2_, pH, oxygen saturation, and hemoglobin fractions^23^.

In the 3PM/ABE model, we applied previously described assumptions for acid-base modelling in HD^12,13^ in the context of PD. Thus, PD treatment was assumed to affect acid-base equilibrium of the body through fluid removal (hemoconcentration) and through the transperitoneal transport of bicarbonate and dissolved CO_2_, which causes changes in their plasma concentration. Assuming homogenous flows in the capillaries of the peritoneum, the concentrations of bicarbonate and dissolved CO_2_ at the venous ends of the peritoneal capillaries (subscript *PD*) can be expressed as:

1 $${C}_{Bic,PD}={C}_{Bic,a}+\frac{dBic}{{\dot{Q}}_{PM}}+\frac{dLact}{{\dot{Q}}_{PM}}$$

2 $${C}_{CO2,PD}={C}_{CO2,a}+\frac{d{CO}_{2}}{{\dot{Q}}_{PM}}$$

where ${C}_{Bic,a}$ and ${C}_{CO2,a}$ are the concentrations at the arterial ends of the capillaries (equal to the concentrations in the arterial blood compartment), and ${\dot{Q}}_{PM}$is the perfusion flow rate across the whole peritoneal membrane (assumed equal to 55 mL/min^25,26^. The terms dBic, dCO_2_ and dLact correspond to the transperitoneal flows of bicarbonate, CO_2_, and lactate, respectively, calculated according to the 3PM equations (positive flows mean transport from dialysate to blood). Supplemented lactate is metabolized in the liver to generate bicarbonate with a 1:1 ratio^27^, which, in the model, is assumed to occur instantly. The concentrations in blood coming out of the peritoneal exchange, ${C}_{Bic,PD}$ and ${C}_{CO2,PD}$, are used to solve again the acid-base model and evaluate the new total carbon dioxide concentration in the buffer-enriched blood, which is then mixed homogeneously with the flow of blood that bypassed the peritoneum, resulting in the mixed-venous blood compartment^12^.

Pre-dialytic total body water and blood volumes were estimated with anthropometric formulas^28^. Water removal was calculated as per 3PM theory, with a constant fraction of total UF removed from the interstitium (20%) and the rest from the vascular compartments; the proportion with which water was removed from arterial and mixed-venous compartments was fixed and equal to their assumed initial volume ratio^24^.

The initial acid-base status of each patient was defined using pre-dialytic measurements of venous bicarbonate plasma concentration, dissolved CO_2_, and base excess, which allowed the calculation of resting metabolic parameters (rate of oxygen consumed and of CO_2_ produced by tissue cells) and minute ventilation, assumed to be constant for the whole simulated dwell. For lack of data, pre-dialysis venous oxygen saturation was assumed equal to 0.65 for all patients. All other physio-chemical parameters connected to the description of the acid-base equilibrium were assumed to be as in previous implementations of the model^12,23,24^. A full description of the model equations is available in the Supplementary Material (Appendices B to E).

### Parameters estimation

In order to tune the model to individual data, we estimated the peritoneal hydraulic conductivity (LpS), its fraction attributed to aquaporins (α_UP_), and scaling factors for the effective permeability surface area coefficients (PS) for each solute (i.e. ratios of the fitted PS value to the PS value predicted by the 3PM). Additionally, for each dwell, the determination of the initial conditions for the acid-base model was carried out via the estimation of the consumption rate of oxygen (${\dot{V}}_{O2,t}$) and production rate of CO_2_ (${\dot{V}}_{CO2,t}$). The estimation was carried out through nonlinear least squares minimization of a cost function (function *lsqnonlin* in MatLab R2024a, The MathWorks Inc., Natick, Massachusetts). The goodness of the fit is reported in this paper as percent root mean squared error (RMSE) relative to the data.

## Results

**Fig. 1:**
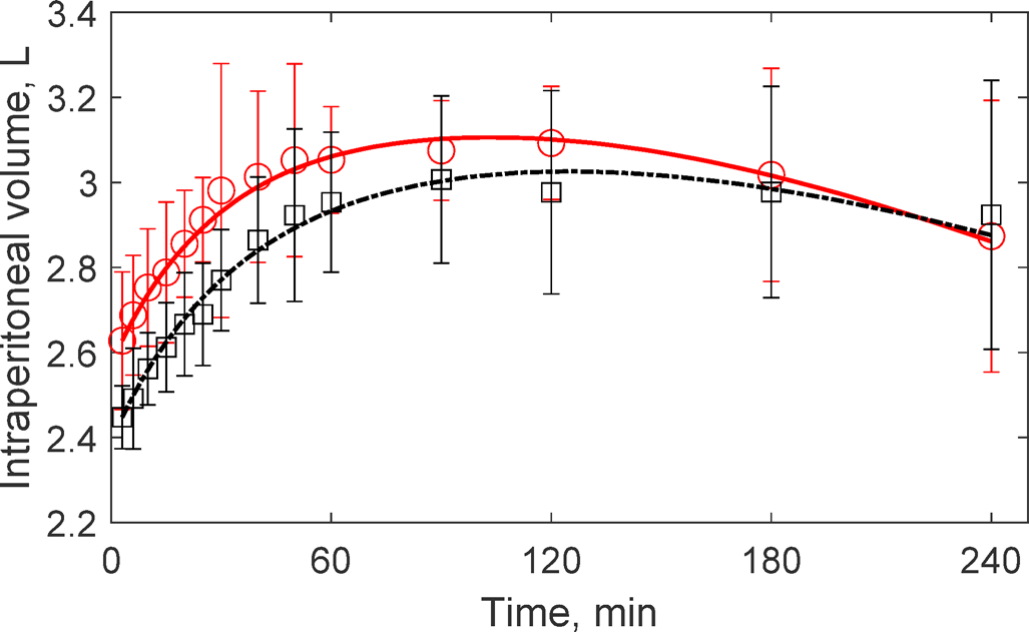
Average value (± SD) of peritoneal fluid volume in B/L versus PD4 dwells. Continuous line and circles—simulation and measured values, respectively, for B/L; dashed line and squares—simulation and measured values, respectively, for PD4.

**Fig. 2:**
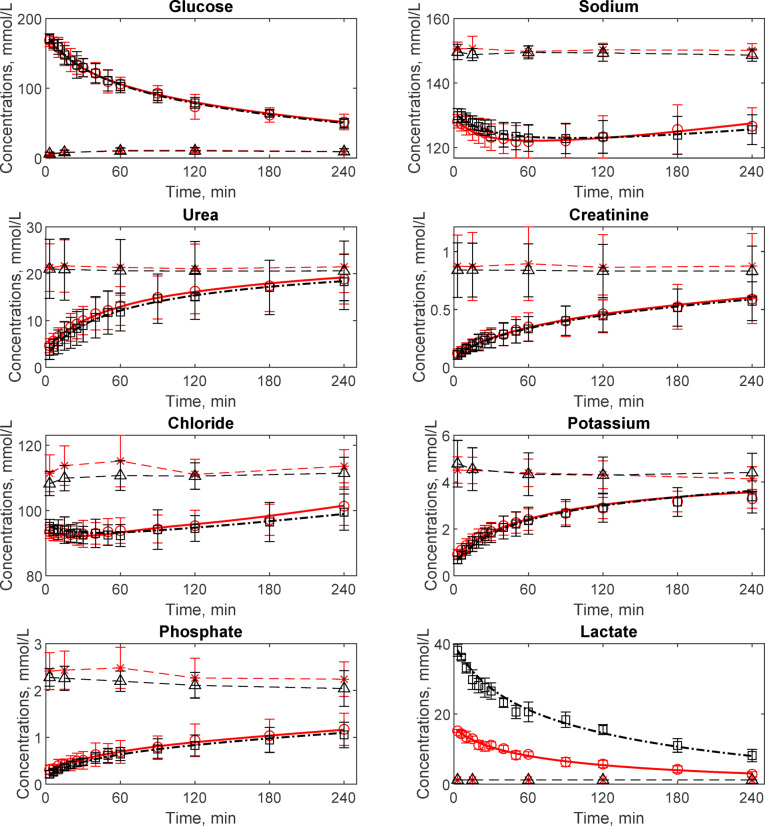
Average values (± SD) of solutes concentrations. Continuous line and circles—simulation and measured values in dialysate, respectively, for B/L; dot-dash line and squares—simulation and measured values in dialysate, respectively, for PD4. Stars—plasma measurements in B/L; triangles—plasma measurements in PD4; dashed lines—interpolated plasma values.

**Fig. 3:**
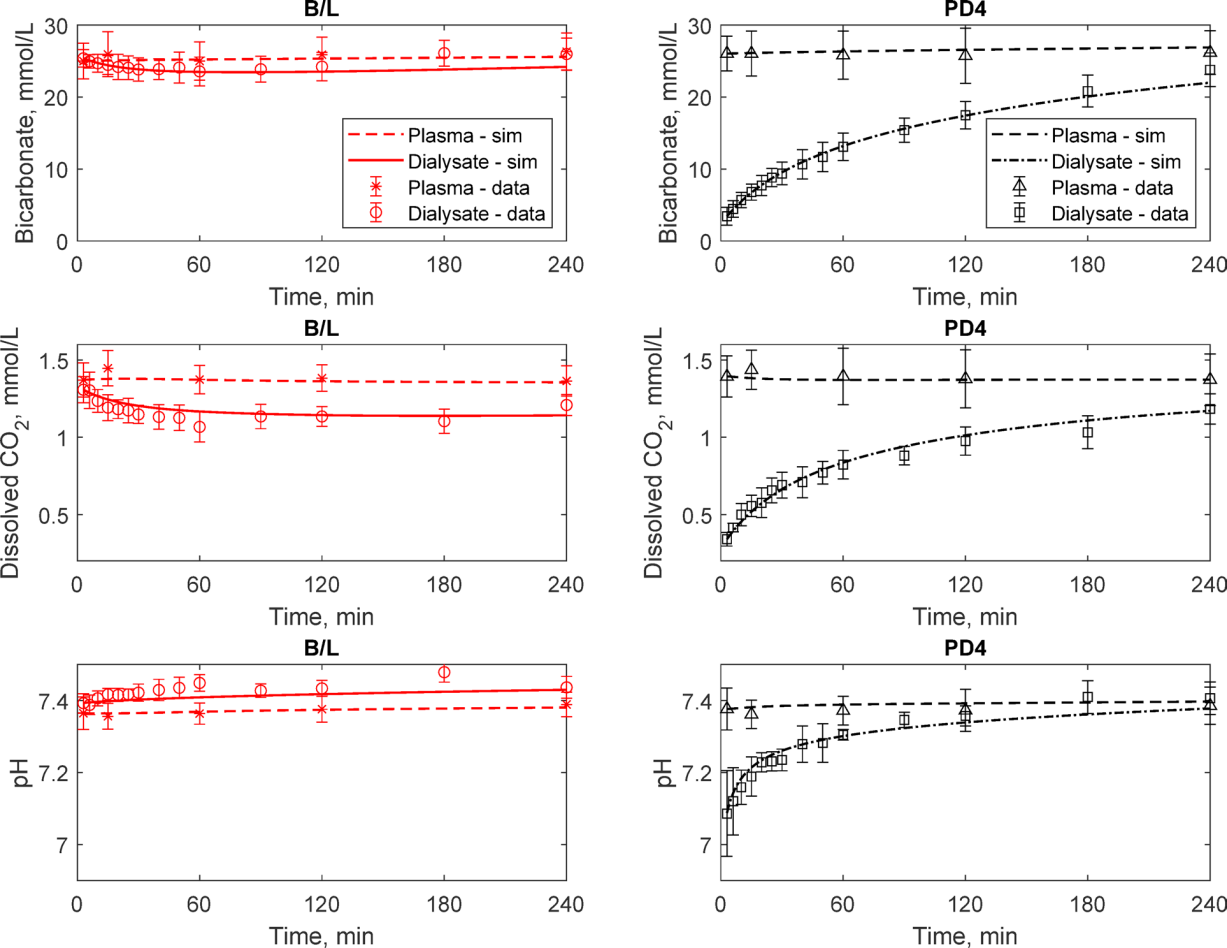
Average values (± SD) of acid-base related variables in plasma and in dialysis fluid.

**Fig. 4:**
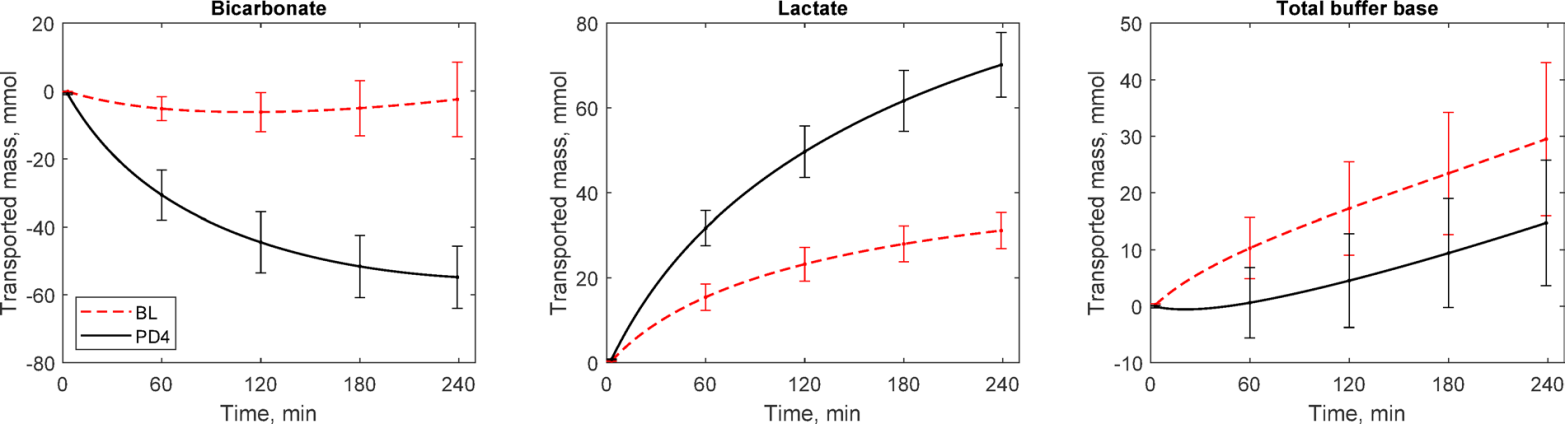
Buffer base change in dialysate during the dwell, predicted by the model for both fluids (average ± SD). The values reported represent the sum of transport rates through the pore system and tissue absorption. Positive values represent transport from dialysate to blood.

**Fig. 5:**
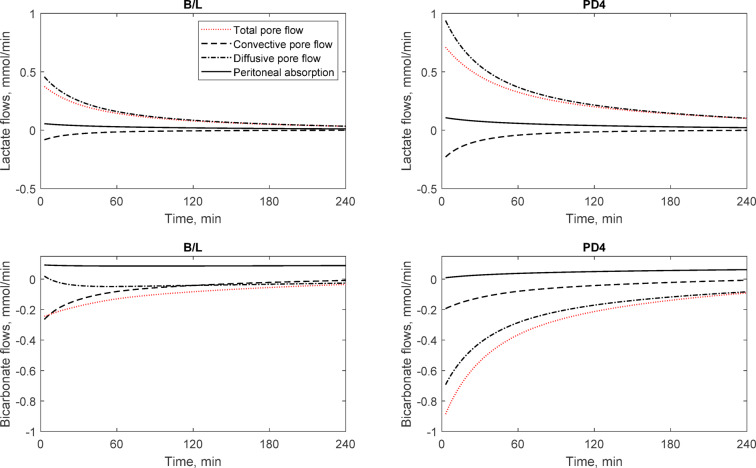
Components of transperitoneal mass flows simulated by the model for bicarbonate and lactate (average profiles, error bars are omitted). Positive values represent transport from dialysate to blood.

The average measured and simulated values of peritoneal fluid volume and small solutes concentration in dialysate are shown in Figs. 1 and 2, respectively, while Fig. 3 displays the acid-base variables in both dialysate and plasma. The different buffer composition of the two fluids was reflected mostly in the different kinetics for lactate, bicarbonate, and dissolved CO_2_, as expected. The almost-physiological concentration of bicarbonate in B/L fluid resulted in a negligible net mass transfer from blood to dialysate, compared to the significant removal observed in PD4 dwells (Fig. 4); with physiologically negligible plasma concentrations, lactate was always delivered to the patient, albeit with different rates. Figure 5 shows the transperitoneal mass flows for bicarbonate and lactate simulated by the model. Because of the higher initial concentration gradient, diffusive transport of bicarbonate was predominant in PD4, compared to B/L dwells in which convection and diffusion have similar magnitude.

Different peritoneal volume kinetics were observed up to 25 min (*p* < 0.05), probably caused by the small differences in residual and infused volumes. Plasma concentrations of solutes were mostly constant during the dwells, only glucose, chloride (PD4 only), and potassium (PD4 only) showed more than one measurement significantly different from the pre-dialytic value (Fig. 2).

According to the classification of transport properties by Twardowski, 1 patient was a high transporter, 3 patients were high-average transporters, and 2 patients were low-average transporters, based on creatine D/P at 4 h^29^. However, no differences in bicarbonate or lactate kinetics were observed in the data based on transporter type.

The fit of the model was in general good. When the model was fitted to the data, the overall root mean squared error (including all dialysate-related measurements) was 6.6 ± 1.4% and 6.4 ± 0.9% for the simulations with B/L and PD4 fluids, respectively (*p* > 0.05). Errors for individual solutes were all less than 10%. Table 1 reports the average values of the parameters estimated to fit the model to dialysate data for each dwell. The two solutions, B/L and PD4, did not differ as regards PS values except for chloride and lactate for which solutes PS values were significantly higher in B/L than in PD4.

**Table 1:** Average values (± SD) of the estimated parameters for the two sets of simulations.

	B/L	PD4
LpS	0.1 ± 0.032	0.11 ± 0.05
α_UP_	0.03 ± 0.015	0.02 ± 0.011
PS Glucose	12.02 ± 3.63	12.23 ± 3.39
PS Sodium	11.06 ± 5.60	6.59 ± 3.16
PS Urea	25.33 ± 5.46	23.03 ± 3.29
PS Creatinine	12.01 ± 3.86	11.45 ± 2.01
PS Chloride	11.35 ± 3.92*	7.74 ± 2.59
PS Potassium	22.75 ± 6.24	20.81 ± 4.23
PS Phosphate	5.18 ± 2.49	5.29 ± 2.07
PS Lactate	19.80 ± 4.92*	15.43 ± 2.66
PS Bicarbonate	28.54 ± 12.34	18.70 ± 4.69
PS CO_2_	20.20 ± 20.67	27.23 ± 7.44

LpS—hydraulic conductivity (mL/min/mmHg); α_UP_—fraction of ultrasmall pores. The value of the permeability coefficients (PS, mL/min) reported is the sum total of small and large pores. *p < 0.05 for B/L versus PD4.

In general, the estimated correction factors for PS values were not significantly different from 1, with the exception of those for chloride (0.96 ± 0.24 for B/L and 0.44 ± 0.15 for PD4), phosphate (0.39 ± 0.18 for B/L and 0.40 ± 0.16 for PD4), urea (1.76 ± 0.38 for B/L and 1.60 ± 0.23 for PD4), and sodium (0.66 ± 0.33 for B/L, with *p* = 0.053, and 0.39 ± 0.19 for PD4). Although not statistically significant, the correction factor for bicarbonate was 1.47 ± 0.64, suggesting that a higher diffusion parameter was required to fit B/L fluid data than that calculated according to the 3-pore theory; by comparison, the average correction factor in PD4 dwells was 0.96 ± 0.24. Correction factors for CO_2_ displayed the highest scattering: 0.81 ± 0.82 in B/L and 1.09 ± 0.30 in PD4, with *p* > 0.05 versus unity in both cases.

The tissue metabolic parameters defining the acid-base pre-dialytic state of each dwell showed on average tendency to be slightly different between fluids; ${\dot{V}}_{O2,t}$ was 0.28 ± 0.01 L/min for B/L versus 0.29 ± 0.01 L/min for PD4 (*p* = 0.054) and ${\dot{V}}_{CO2,t}$ was 0.19 ± 0.03 L/min for B/L versus 0.16 ± 0.016 L/min for PD4 (*p* = 0.050). They resulted however in a significantly higher resting minute ventilation for B/L, 3.52 ± 0.73 L/min versus 2.83 ± 0.296 for PD4 (*p* = 0.039).

Plasma concentrations of bicarbonate and dissolved CO_2_ were predicted with a RMSE of 4.0 ± 2.8% and 7.2 ± 5.3% for B/L, and 5.7 ± 4.4% and 5.2 ± 3.5% for PD4 (*p* > 0.05 between fluids).

The model respiration parameters, by default assumed constant during a dwell, can be modified to simulate different scenarios. For example, Fig. 7 shows CO_2_ data and simulations of a patient with similar patterns in both the B/L and PD4 dwells. The details of these profiles could only be modelled by introducing changes in minute ventilation during the dwell, as described in the figure.

## Discussion

We used a comprehensive whole-body model of total carbon dioxide transport to investigate how different buffer compositions in dialysis fluids affect acid–base balance during peritoneal dialysis dwells. Peritoneal buffer transport and acidosis correction during peritoneal dialysis were analyzed based on data from two PD dwells, one with acidic lactate-based dialysis fluid (B/L) and one with a mix of bicarbonate and lactate with close to physiological pH (PD4).

The model we used was originally proposed for hemodialysis^12^, but was adapted with few modifications, mainly involving the use of the 3-pore model of peritoneal transport to describe the exchange of water and small solutes between dialysis fluid and plasma. The transport characteristics of the peritoneal membrane, namely the hydraulic conductivity (LpS) and the fraction of aquaporins transport (α_UP_), along with a corrective factor for the membrane permeability of each of the ten small solutes described by the model, were estimated to achieve the best fit to the clinical data. This resulted in an accurate simulation of the transport kinetics for all substances (Figs. 1 and 2) with low prediction errors. Although similar predictions of dialysate concentration profiles can be achieved using the 3-pore theory alone, the implementation of the new 3PM/ABE model allowed us to additionally predict the changes of bicarbonate, dissolved CO_2_, and pH in plasma (Fig. 3). Modelling transport alone is not sufficient to describe changes to blood bicarbonate and CO_2_ levels during dialysis, due to the complex nature of the bicarbonate buffer equilibrium, which involves an open loop system through the alveolar exchange of gaseous CO_2_; thus, the amount of bicarbonate that appears in the blood is not necessarily equal to the amount that disappears from dialysis fluid. A comprehensive model implementing the regulation of both fixed and volatile acids is necessary for a truly physiological description of acid-base balance.

In general, the results of the applied 3PM/ABE model show: (1) that the model adequately describes the kinetics of solutes including the used buffers, lactate and lactate/bicarbonate respectively, and (2) that – in spite of the intended differences in dialysate concentrations of buffers and dialysate pH – the diffusive transport parameters in general were similar for the two solutions with the exception of higher PS values with the B/L solution for lactate and chloride.

The differences in the estimated transport parameters between B/L and PD4 were largely negligible (Table 1), and the different composition of the two dialysis solutions was reflected almost exclusively in the different kinetics of bicarbonate, dissolved CO_2_, and lactate (Figs. 2 and 3). Concentration profiles behaved as expected given the initial differences in bicarbonate concentration gradients between the two buffer solutions. Bicarbonate was in both cases removed through the peritoneum from circulation, although with a significantly higher rate with PD4 (and mostly through diffusion), for a total 65.7 ± 10.8 mmol removed with PD4 vs. 23.1 ± 6.8 mmol with B/L (excluding mass regained through absorption by the tissue). This removal was compensated by the higher delivery of lactate in PD4, and its consequent conversion to bicarbonate. Because of this mechanism, notwithstanding the very different kinetics generated by the two buffer fluids, described in Figs. 4 and 5, the model predicted an almost constant bicarbonate concentration profile in plasma, as observed in the clinical data.

**Fig. 6:**
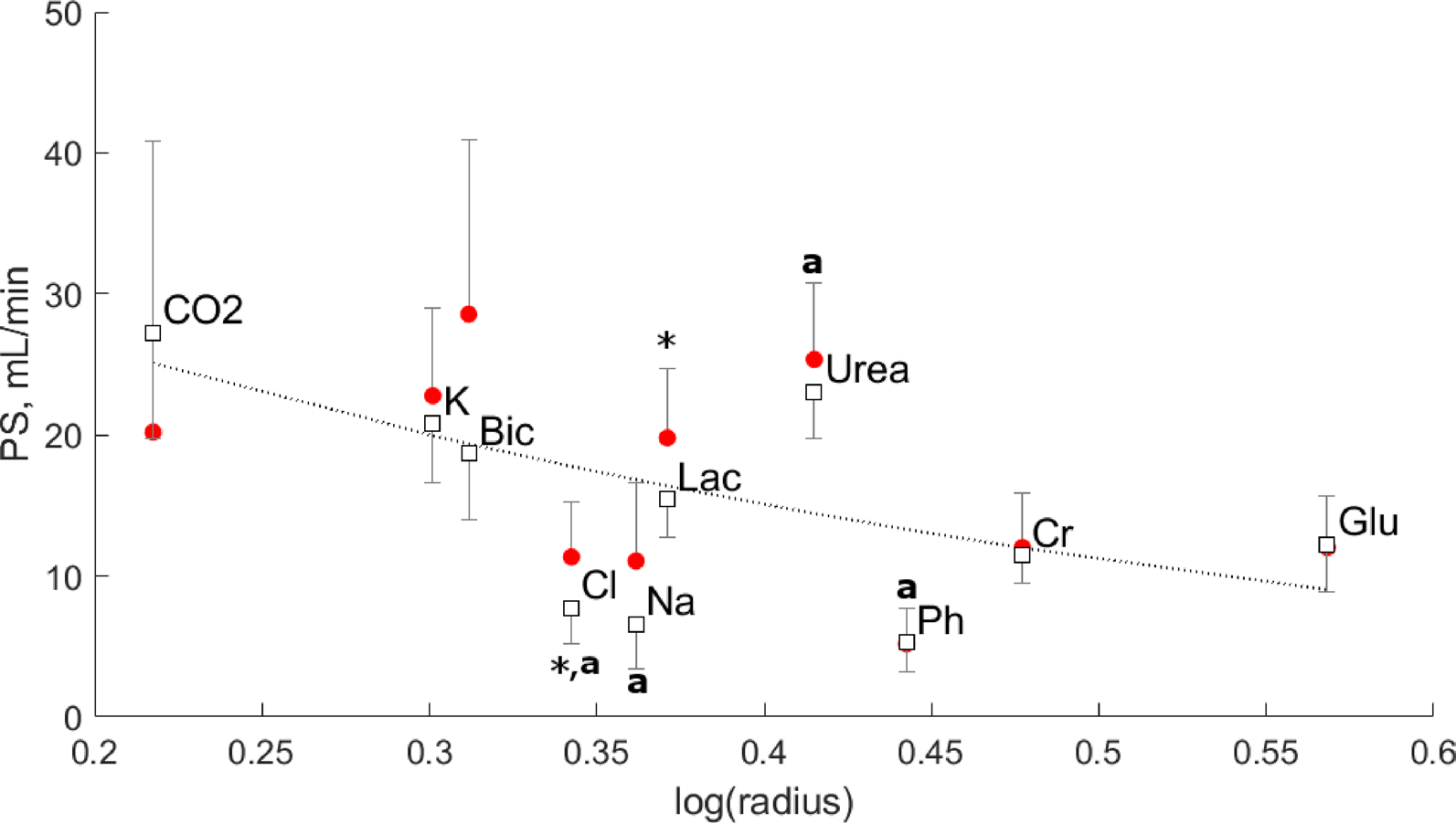
Scatterplot illustrating the relationship between molecular radii of solutes (in Å) and average values of the permeability-surface are products (PS) estimated by the model (red circles – B/L; white squares – PD4). The values calculated according to the 3-pore theory alone, without the estimated correction factors, lay on the dotted line. * *p* < 0.05 for B/L vs. PD4. ^a^ *p* < 0.05 vs. theoretical values.

The correction factors estimated for the permeability surface area products (PS) were consistent with previous modelling studies, in particular with regards to the need to increase coefficients for urea permeability and reduce sodium’s to fit the data^19^. The high scattering of PS for bicarbonate and CO_2_ estimated in B/L dwells (Table 1) was likely caused by these solutes being close to equilibrium between dialysate and blood, making it difficult to unambiguously estimate a single optimal value of PS. Although with some variability, the average corrected PS values reflected, for the most, the relationship with molecular radius described by the standard 3PM (Fig. 6). PS values for phosphate were significantly lower than could be expected from its molecular radius and weight. Measurements of phosphate report total inorganic phosphate, of which diffusible ionic phosphate is the major fraction, but protein-bound phosphate would not participate in peritoneal transport. Correcting the measured values to equal only diffusible phosphate would likely provide more accurate estimation of PS for this solute^30^. As expected, PS for urea was higher than predicted by the 3PM theory; this could result from additional diffusion of urea through cellular membranes, which was not accounted for by the model^25,31^.

Sow et al.^32^ reported from experimental PD studies in mice, that bicarbonate was locally produced in the peritoneal membrane by carbonic anhydrase, a process that could be blocked by a carbonic anhydrase inhibitor. However, in the present study, we could not identify a significant local intraperitoneal production of bicarbonate.

**Fig. 7:**
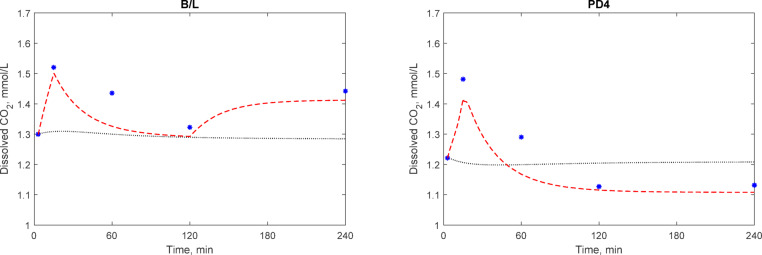
Concentration of CO_2_ dissolved in plasma measured in both dwells of the same patient (stars). With constant minute ventilation calculated from the pre-dialytic bicarbonate and pCO_2_ data, the model predicted a flat profile (dotted lines). Dashed lines represent the simulations with variable minute ventilation. The profile observed with B/L could be approximated, assuming a 35% decrease in minute ventilation for 15 min, followed by a return to the pre-dialytic value until 120 min, after which minute ventilation decreased again by 10%. The PD4 profile was described assuming a 55% decrease in minute ventilation for 15 min, followed by a 10% increase compared to the initial value until the end of the dwell.

Our results demonstrate that the 3PM/ABE model is able to predict the plasma response to acidosis correction with good accuracy, using a set of previously described standard acid–base parameters for all patients^23^. The assumption of constant metabolic and respiratory parameters throughout the dwell did not, on average, significantly reduce the overall quality of the fit. However, some variability in plasma kinetics was observed, particularly in CO_2_ profiles, some of which could not be fully captured under these assumptions. Dynamic changes in the model parameters, such as those in the example simulations showed in Fig. 7, may reflect impaired respiration due to increased intra-abdominal pressure from peritoneal filling, which subsequently declines as the body adapts to the infusion of dialysis fluid. We previously showed that assuming a variable rate of respiration improved the descriptions of HD data during a single session^12^ but also was required to fit the simulation to data recorded throughout a weekly cycle of dialysis^33^. The pre-dialytic minute ventilation values, B/L 3.5 vs. PD4 2.8 L/min, that were estimated by the 3PM/ABE model for the patients in the present study (Table 1) are significantly lower compared to the average for a healthy human (5–8 L/min^34^, . These values could be indicative of respiratory impairment correlated with acidosis, but also the estimation is affected by the simplifications used as regards respiratory regulation mechanisms introduced in the model, whose focus was primarily the biochemistry of the buffer equilibrium^23^. Cases such as those exemplified in Fig. 7 highlight the model’s capacity to accommodate diverse and specific patterns and underline the importance of a comprehensive description of both transport kinetics and respiratory control in explaining the perturbations to acid-base homeostasis.

In addition to the assumption of constant respiration parameters, the model has several other limitations. The representation of non-reactive solutes in the body is significantly simplified compared to the modelling of the components of the bicarbonate buffer. Transperitoneal transport was modelled to fit dialysate-side solute kinetics in order to determine osmotic gradients, with plasma concentrations assumed known from data and thus independent from transport. This simplification was deemed justifiable by the negligible changes in the plasma concentrations of small solutes routinely observed during PD, and by the need to reduce the computational load required to solve the model. The drawback is not accounting for the movement of solutes between plasma, tissue cells and erythrocytes, therefore ignoring the coupling of bicarbonate, lactate, and other ions in the preservation of the electroneutrality principle. The original acid-base model at the core of our study was based on the “Copenhagen” approach to the description of acid-base disorders popularized by Siggaard-Andersen^35^, according to which the nature of acidosis can be determined from changes to base excess and partial pressure of CO_2_ from the norm; pH and bicarbonate are treated as derived values influenced by those changes. A simpler model to describe changes in bicarbonate concentration during PD was proposed by Wolf^11^, using an alternative approach focused on electroneutrality considerations (the “Stewart” approach^36^, in which bicarbonate and H^+^ concentrations are derivative of the strong ion difference and non-bicarbonate buffers concentration); Wolf’s model was able to fit bicarbonate profiles in dialysate but offered no insight on the acid-base response in the body. Although every modeling approach introduces some degree of approximation error, both Stewart and Copenhagen methods should lead to similar results, being different lenses used to investigate the same physical phenomena. In the case of models based on the Copenhagen approach, such as the one here described, neglecting the implications of the electroneutrality principle could have resulted in the observed discrepancies between measured and predicted pH values (Fig. 3), the need for significant PS correction factors for chloride, sodium, urea, and phosphate, as well as the large standard errors in the PS values for bicarbonate and CO_2_. Future efforts with this model should be directed to include electroneutrality constraints in the solution of the acid-base chemistry equations for each compartment. Another significant simplification regarded the effect of lactate supplementation on the acid-base regulation. Lactate was assumed to be converted to bicarbonate upon crossing the peritoneal membrane with a 1:1 ratio; the new bicarbonate appears in the model instantaneously at the venous ending of the peritoneal capillaries, setting aside delays caused by the conversion being localized in the liver or by the rate at which bicarbonate is produced. It must be however noted that the patients were treated continuously with Dianeal PD4 even before participating in the study, therefore it is reasonable to assume that the conversion process of lactate was already ongoing during the two dwells described with our model. If anything, we could have expected changes in plasma lactate levels during the B/L dwells, but such differences were not observed in data or simulations. Lastly, to easily integrate the 3PM with the new 3PM/ABE model, we assumed that the transport characteristics of the peritoneum could be described with a homogeneous and constant rate of blood perfusion, whose value was based on the available literature.

This was an exploratory study to validate a new version of our previous model of acidosis correction in HD, modified to describe PD dwells. The model was able to reproduce the bicarbonate buffer response to base supplementation observed in PD data. Although changes in plasma were small, we were able to simulate specific patterns of plasma CO_2_ concentrations applying additional assumptions on the intradialytic changes to the respiration rate. In conclusion, when the model was applied to simulate dwells with acidic or neutral dialysis fluid, the buffer composition did not substantially affect the estimation of the peritoneal transport characteristics for water and bicarbonate, nor for other solutes (with the exception of dialysate chloride and lactate). The acid-base regulatory response codified in the model worked as expected regardless of the composition of the dialysis solution.

## Supplementary Information

Below is the link to the electronic supplementary material.

Supplementary Material 1

## Data Availability

The data is available upon reasonable request.
