# Supplementary material for: Mathematical modeling of peritoneal buffer transport and acidosis correction in patients on peritoneal dialysis
Source: Sci Rep. 2026 Jul 4;16:20555. doi: 10.1038/s41598-026-53800-0 (PMC13333014; doi:10.1038/s41598-026-53800-0)
Supplement: Supplementary file 1 — Supplementary Material 1 [file 41598_2026_53800_MOESM1_ESM.docx]

Supplementary Material

to

Mathematical modeling of peritoneal buffer transport and acidosis correction in patients on peritoneal dialysis

Pietribiasi M., Stachowska-Pietka J., Waniewski J., Lindholm B., Heimbürger O.

Appendix A

**Table A1.** Parameters of the 3-pore model [1,2].

| Parameter | Value |
| --- | --- |
| A_0_/Δx | 25000 cm |
| α_LP_ | 8% |
| dP_0_ | 8 mmHg |
| dΠ_i_ | 22 mmHg |
| r_LP_ | 250 Å |
| r_SP_ | 43 Å |
| r_urea_ | 2.60 Å |
| r_glucose_ | 3.70 Å |
| r_sodium_ | 2.30 Å |
| r_creatinine_ | 3.00 Å |
| r_proteins_ | 35.50 Å |
| r_chloride_ | 2.20 Å |
| r_potassium_ | 2.00 Å |
| r_phosphate_ | 2.77 Å |
| r_CO2_ | 1.65 Å |
| r_bicarbonate_ | 2.05 Å |
| r_lactate_ | 2.35 Å |

A_0_/Δx – effective free diffusion surface area over diffusion path length; α_LP_ – fractional hydraulic conductivity of large pores; dP_0_ – initial hydraulic pressure gradient; dΠ_i_ – oncotic pressure gradient; r – radius.

Appendix B.

**Acid-base status equations for blood capillaries**

The following system of equations is described in detail in [3], and here is given only a condensed formulation. Supplementary Equations S1 – S20 can be solved simultaneously given a combination of known variables obtained from the fixed parameters of the model and solution of the state equations.

*Symbols*

*tCO_2_* – total CO_2_ concentration (mmol/L).

*tO_2_* – total oxygen concentration (mmol/L).

*pCO_2_* – CO_2_ partial pressure (kPa).

*pO_2_* – oxygen partial pressure (kPa).

*Bic* – bicarbonate concentration (mmol/L).

*Hct* – haematocrit.

*σ_CO2_ , σ_O2_* – CO_2_, O_2_ solubility (mmol/L/kPa).

$HBNHCO_{2}^{-}$, $HbO_{2}NHCO_{2}^{-}$, $HbNH_{3}^{+}$, $HbO_{2}NH_{3}^{+}$, $HbNH_{2}$, $HbO_{2}NH_{2}$ - molecular variants of hemoglobin amino end (mmol/L).

$Hb(RH)_{b}$, $Hb(R^{-})_{b}$, $HbO_{2}(RH)_{b}$, $HbO_{2}(R^{-})_{b}$ - molecular variants of hemoglobin side chains (mmol/L).

*Hb_e_* – total hemoglobin concentration (mmol/L).

*sO_2_* – oxygen saturation.

$\Psi_{37^{\circ}}$ - oxygen saturation at 37C, as a function of pH_p_, pO_2_, and pCO_2_ [4].

*HNBBp*, $NBB_{p}^{-}$ - molecular variants of non-bicarbonate buffer base (mEq/L).

*BE* – base excess (mmol/L)

*nBB* – total buffer base under standard conditions (mmol/L) [5].

*pK* – acid dissociation constant.

*Subscripts*

*p* – plasma

*e* – erythrocytes

*Equations*

$$tCO_{2}=\left(\sigma_{CO2,p}pCO_{2}+Bic_{p}\right)\left(1-Hct\right)+\left(\sigma_{CO2,e}pCO_{2}+Bic_{e}+HbNHCO_{2}^{-}+HbO_{2}NHCO_{2}^{-}\right)Hct$$

$$tO_{2}=\sigma_{O2,p}pO_{2}\left(1-Hct\right)+\left(\sigma_{O2,e}pO_{2}+sO_{2}Hb_{e}\right)Hct$$

$$Hb_{e}=HbNH_{3}^{+}+HbNH_{2}+HbNHCO_{2}^{-}+HbO_{2}NH_{3}^{+}+HbO_{2}NH_{2}+HbO_{2}NHCO_{2}^{-}$$

$$Hb_{e}=Hb(RH)_{b}+Hb(R^{-})_{b}+HbO_{2}(RH)_{b}+HbO_{2}(R^{-})_{b}$$

$$sO_{2}=\left(HbO_{2}NH_{3}^{+}+HbO_{2}NH_{2}+HbO_{2}NHCO_{2}^{-}\right)/Hb_{e}$$

$$sO_{2}=\left(HbO_{2}(RH)_{b}+HbO_{2}(R^{-})_{b}\right)/Hb_{e}$$

$$sO_{2}=\Psi_{37^{\circ}}\left(pH_{p},pO_{2},pCO_{2}\right)$$

$$pH_{e}=7.19+0.77(pH_{p}-7.4)+0.031\left(1-sO_{2}\right)$$

$$tNBB_{p}=HNBB_{p}+NBB^{-}_{p}$$

$$\begin{matrix} BE=\left(Bic_{p}+NBB_{p}^{-}\right)\left(1-Hct\right) \\ +\left[Bic_{e}+b\left(Hb(R^{-})_{b}\right)+b\left(HbO_{2}(R^{-})_{b}\right)+HbNH_{2}+HbO_{2}NH_{2}\right]Hct \\ +\left[2\left(HbNHCO_{2}^{-}\right)+2\left(HbO_{2}NHCO_{2}^{-}\right)\right]Hct-nBB \end{matrix}$$

$nBB=41.7\left(1-Hct\right)+72.5\cdot Hct$[5]

two mass action equations for plasma pH and 7 for erythrocytes pH are written, one for each pair of weak acid *HA* and base *A^-^* (CO_2_ and bicarbonate, non-bicarbonate buffer and hemoglobin dissociated forms):

$pH=pK_{A}+\log\left(\frac{A^{-}}{HA}\right)$ - (S20)

Appendix C.

**Acid-base status equations for interstitial fluid and tissue cells**

Supplementary Equations S21 – S30 are solved simultaneously to give the value of variables describing the interstitial fluid and tissue cells acid-base status. The system is described in detail in another paper [6].

*Symbols*

*V* – volume (L).

– sensitivity of intracellular pH to changes in interstitial pH.

$\beta_{NBBt}$ – buffer capacity of non-bicarbonate tissue buffer (mmol/L/pH).

*nBB_i_* – normal total interstitial buffer base [6]

*npH_i_* – normal interstitial pH [6]

*nBic_t_* – normal tissue cells bicarbonate concentration [6]

*npH_t_* – normal tissue cells pH [6]

*Subscripts*

*it* – average of interstitial fluid and tissue cells.

*i* – interstitial.

*t* – tissue cells.

*Equations*

$tCO_{2,it}=\frac{\left(Bic_{i}+\sigma_{CO2,i}pCO_{2,i}\right)V_{i}+\left(Bic_{t}+\sigma_{CO2,t}pCO_{2,t}\right)V_{t}}{V_{i}+V_{t}}$ (S21)

$pCO_{2,i}=pCO_{2,t}$ (S22)

$tNBB_{i}=HNBB_{i}+NBB_{i}^{-}$ (S23)

$BE_{it}=\frac{BE_{i}V_{i}+BE_{t}V_{t}}{V_{i}+V_{t}}$ (S24)

$BE_{i}=Bic_{i}+NBB_{i}^{-}-nBB_{i}$ (S25)

$BE_{t}=Bic_{t}-nBic_{t}+\beta_{NBBt}\left(pH_{t}-npH_{t}\right)$ (S26)

$pH_{i}=pK_{Bic,i}+\log\left(\frac{Bic_{i}}{\sigma_{CO2,i}pCO_{2,i}}\right)$ (S27)

$pH_{i}=pK_{NBB,i}+\log\left(\frac{NBB_{i}^{-}}{HNBB_{i}}\right)$ (S28)

$pH_{t}=pK_{Bic,t}+\log\left(\frac{Bic_{t}}{\sigma_{Bic,t}pCO_{2,t}}\right)$ (S29)

(S30)

Appendix D.

*Symbols*

*FeCO_2_, FeO_2_* – expired fractions of CO_2_, O_2_.

*FiCO_2_, FiO_2_* – inspired fractions of CO_2_, O_2_.

– cardiac output (L/min).

*V̇_E_* – minute ventilation (L/min).

*p_b_ – environment barometric pressure (mmHg).*

*p_H2O_ - pressure of saturated water vapor at 37C (mmHg).*

*V* – volume (L).

*tCO_2_, tO_2_* – total carbon dioxide, total oxygen (mmol/L).

*BE* – base excess (mmol/L).

*V̇CO_2,t_* - tissue production of CO_2_.

*H_t_* – net acid production rate (mmol/min).

*Subscripts*

*A – lung alveoli.*

*l – lung capillary.*

*a – arterial.*

*mv – mixed-venous output.*

*mv,in – mixed-venous input.*

*v – venous.*

*vp – venous end of the peritoneal capillaries.*


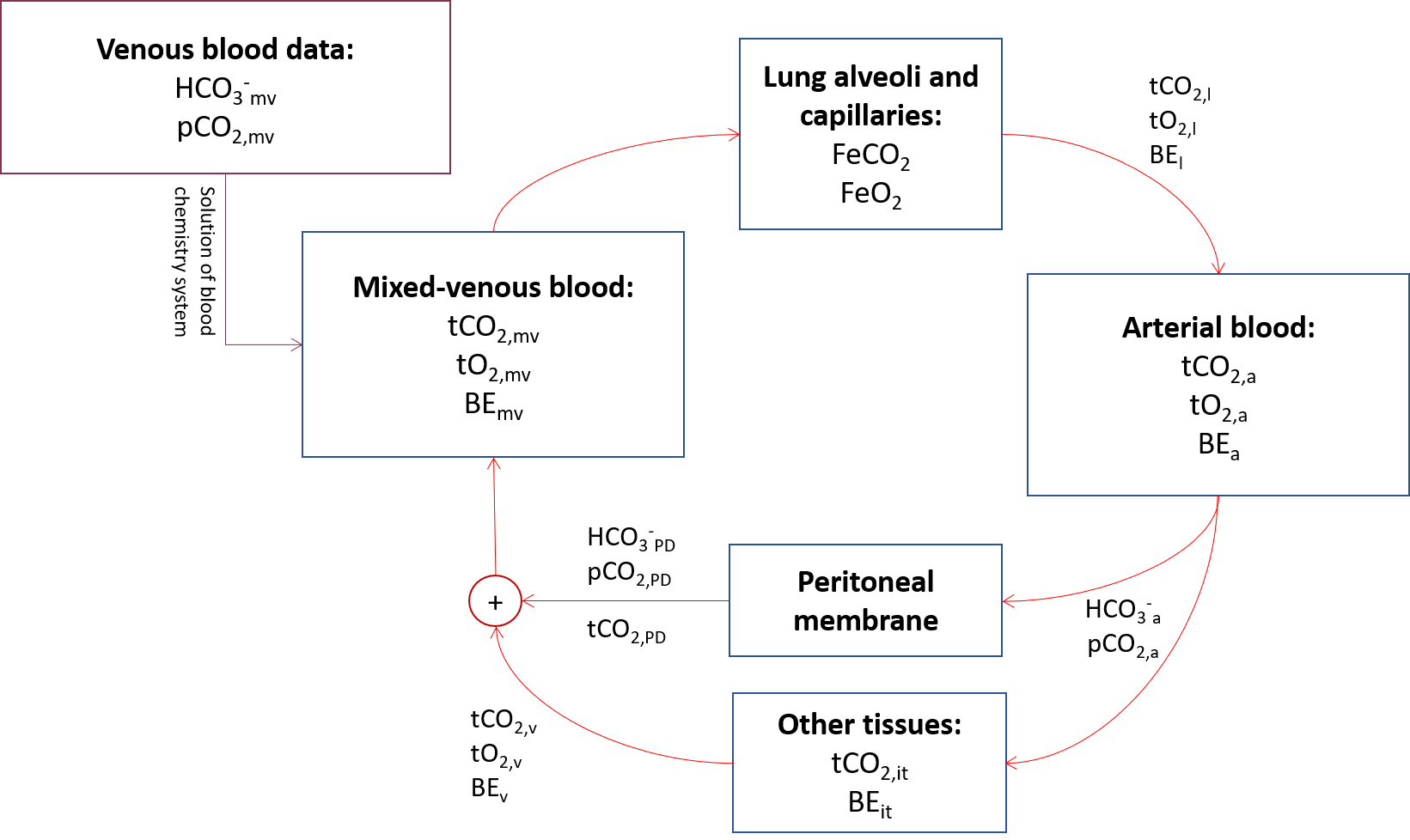


**Figure D1.** Structure of the whole-body model of CO_2_ and O_2_ transport during peritoneal dialysis. Subscript ‘PD’ refers to the base-enriched blood coming out of the peritoneal exchange (see Equations 1 and 2 in the main text).

**Time-dependent equations of the model**

The state equations of the model are given in a condensed form, the detailed description can be found in reference [6].

*Lung alveoli and capillaries equations*

(S31)

$\begin{matrix} pCO_{2,l}=pCO_{2,A}=FeCO_{2}\left(p_{b}-p_{H2O}\right) \\ pO_{2,l}=pO_{2,A}=FeO_{2}\left(p_{b}-p_{H2O}\right) \end{matrix}$ (S32)

$BE_{l}=BE_{mv}$ (S33)

*BE_l_*, *pCO_2,l_* and *pO_2,l_* are used to solve the system for blood in the lung capillaries (Appendix B), calculating *tCO_2,l_* and *tO_2,l_*.

*Arterial capillaries equations*

(S34)

*Tissue capillaries, interstitial fluid and tissue cells equations*

*tCO_2,it_* and *BE_it_* are used to solve the system for interstitial fluid and tissue cells (Appendix C), calculating $pCO_{2,i}=pCO_{2,v}$ and $Bic_{i}=Bic_{v}$where the subscript *v* indicates venous blood immediately after the exchange with the tissues. These, together with are used to solve the system (Appendix C) for the venous capillaries, giving *tCO_2,v_* and *BE_v_*.

(S35)

*Venous peritoneal blood and mixed-venous capillaries equations*

*tCO_2,a_*, *tO_2,a_* and *BE_a_* are used to solve the system for arterial capillaries (Appendix B), to calculate C_Bic,a_ and C_CO2,a_ used in the Equations 1 and 2 in the main text to calculate the composition of the blood at the venous ending of the peritoneal capillaries, which has been enriched in buffer by the exchange with dialysis fluid (subscript *PD*). This is mixed with the venous blood flow that bypassed the peritoneum to obtain the composition of the blood entering the mixed-venous compartment:

$$t{CO}_{2,mv,in}=t{CO}_{2,v}\left( 1-\frac{\dot{Q}_{PM}}{\dot{Q}_{CO}} \right)+t{CO}_{2,PD}\left( \frac{\dot{Q}_{PM}}{\dot{Q}_{CO}} \right)$$

where $\dot{Q}_{PM}$ is the blood flow perfusing the peritoneum. Similar equations are written for *tO_2,mv,in_* and *BE_mv,in_*. The state equations for the mixed venous compartment are then:

$\frac{d}{dt}\left( {tCO}_{2,mv} \right)=\frac{\dot{Q}_{CO}}{V_{mv}}\left( {tCO}_{2,mv,in}-{tCO}_{2,mv} \right)$

$\frac{d}{dt}\left( {tO}_{2,mv} \right)=\frac{\dot{Q}_{CO}}{V_{mv}}\left( {tO}_{2,mv,in}-{tO}_{2,mv} \right)$ (S36)

$\frac{d}{dt}\left( {BE}_{mv} \right)=\frac{\dot{Q}_{CO}}{V_{mv}}\left( {BE}_{mv,in}-{BE}_{mv} \right)$

Appendix E.

**Solution of the pre-dialysis steady-state model**

Clinical values of venous plasma bicarbonate concentration and pCO_2_ were taken from the clinical measurements. To complete the initial input to the model a third variable is needed^4^: for lack of data, the value of arterial oxygen saturation was assumed equal to 0.65. sO_2,a_ was chosen because it is usually measured in a clinical setting; however, any other variable describing the acid-base status of blood (Appendix B) could be used. From these three inputs the system of equations describing the venous blood biochemistry was solved univocally. Solving the steady-state versions of the equations presented in Appendix D is then possible to calculate the partial pressures *pCO_2,l_* and *pO_2,l_* in the lung compartment, from which the fractions of the expired CO_2_ (*FeCO_2_*) and oxygen (*FeO_2_*) are calculated:

$FeCO_{2}=\frac{pCO_{2,l}}{p_{b}-pH2O}$ (S37)

$FeO_{2}=\frac{pO_{2,l}}{p_{b}-p_{H20}}$ (S38)

Parameters *p_b_* and *p_H20_* are the barometric pressure and the pressure of saturated water vapor at 37 C, respectively.

Before the start of dialysis, the gas flows between environment and lungs, *V̇CO_2,Ae_* and *V̇O_2,eA_*, are assumed to be in equilibrium with the tissue production of CO_2_ (*V̇CO_2,t_*) and consumption of O_2_ (*V̇O_2,t_*), respectively:

(S39)

(S40)

(S41)

where *V̇_E_* is the minute ventilation, *f* the respiration rate, *V_T_* the pulmonary tidal volume, and *V_D_* the dead volume. Tidal volume was calculated as a fraction of body weight, assuming a physiological normalized tidal volume of 8 ml/Kg body weight; dead volume was considered equal to one third of tidal volume. *FiCO_2_* and *FiO_2_* are constants representing the fractions of inspired CO_2_ and O_2_, respectively. It follows that:

${\dot{\text{V}}}_{\text{E,CO2}}\text{=}\frac{\dot{\text{V}}\text{C}\text{O}_{\text{2,t}}}{\left( \text{FeC}\text{O}_{2}\text{-}\text{FiC}\text{O}_{2} \right)}$ (S42)

${\dot{\text{V}}}_{\text{E,O2}}\text{=}\frac{\dot{\text{V}}\text{O}_{\text{2,t}}}{\left( \text{Fi}\text{O}_{2}\text{-Fe}\text{O}_{2} \right)}$ (S43)

The function:

${f=\dot{V}}_{E,CO2}-\dot{V}_{E,O2}$ (S44)

was minimized via least square optimization, estimating the values of *V̇CO_2,t_* and *V̇O_2,t_* so that, given the input bicarbonate, *pCO_2,a_* and *sO_2,a_*, the values of *V̇_E_* calculated from Equations S42 and S43 would be equal. The two parameters thus identified are then treated as constants for the simulation of the PD dwell. The remaining values necessary to calculate the initial, pre-dialysis state of the model, are obtained simply imposing the steady state of the state equations as described in Appendix B and reference [6].

**References**

[1] Rippe B, Venturoli D, Simonsen O, de Arteaga J. Fluid and electrolyte transport across the peritoneal membrane during CAPD according to the three-pore model. Peritoneal Dialysis International 2004;24:10–27. https://doi.org/10.1177/089686080402400102.

[2] Stachowska-Pietka J, Naumnik B, Suchowierska E, Gomez R, Waniewski J, Lindholm B. Water removal during automated peritoneal dialysis assessed by remote patient monitoring and modelling of peritoneal tissue hydration. Sci Rep 2021;11:1–10. https://doi.org/10.1038/S41598-021-95001-X.

[3] Rees SE, Andreassen S. Mathematical models of oxygen and carbon dioxide storage and transport: the acid-base chemistry of blood. Crit Rev Biomed Eng 2005;33:209–64. https://doi.org/10.1615/CRITREVBIOMEDENG.V33.I3.10.

[4] Siggaard-Andersen O, Wimberley PD, Gothgen I, Siggaard-Andersen M. A mathematical model of the hemoglobin-oxygen dissociation curve of human blood and of the oxygen partial pressure as a function of temperature. Clin Chem 1984;30:1646–51. https://doi.org/10.1093/CLINCHEM/30.10.1646.

[5] Siggaard-Andersen O. The acid-base status of the blood. Acid-base biochemistry, Copenhagen: Williams & Wilkins Company; 1974.

[6] Andreassen S, Rees SE. Mathematical models of oxygen and carbon dioxide storage and transport: interstitial fluid and tissue stores and whole-body transport. Crit Rev Biomed Eng 2005;33:265–98. https://doi.org/10.1615/CRITREVBIOMEDENG.V33.I3.20.
